# V5 and GFP Tagging of Viral Gene pp38 of Marek’s Disease Vaccine Strain CVI988 Using CRISPR/Cas9 Editing

**DOI:** 10.3390/v14020436

**Published:** 2022-02-21

**Authors:** Weicheng Li, Yaoyao Zhang, Katy Moffat, Venugopal Nair, Yongxiu Yao

**Affiliations:** 1 Viral Oncogenesis Group, The Pirbright Institute, Pirbright, Guildford GU24 0NF, UK; weicheng.li@pirbright.ac.uk (W.L.); yaoyao.zhang@pirbright.ac.uk (Y.Z.); kathryn.moffat@pirbright.ac.uk (K.M.); 2 The Jenner Institute Laboratories, University of Oxford, Oxford OX1 4BH, UK; 3 Department of Zoology, University of Oxford, Oxford OX1 4BH, UK

**Keywords:** CRISPR/Cas9, MDV, pp38, tagging, V5, GFP

## Abstract

Marek’s disease virus (MDV) is a member of alphaherpesviruses associated with Marek’s disease, a highly contagious neoplastic disease in chickens. The availability of the complete sequence of the viral genome allowed for the identification of major genes associated with pathogenicity using different techniques, such as bacterial artificial chromosome (BAC) mutagenesis and the recent powerful clustered regularly interspaced short palindromic repeats (CRISPR)/CRISPR-associated protein 9 (Cas9)-based editing system. Thus far, most studies on MDV genome editing using the CRISPR/Cas9 system have focused on gene deletion. However, analysis of the expression and interactions of the viral proteins during virus replication in infected cells and tumor cells is also important for studying its role in MDV pathogenesis. The unavailability of antibodies against most of the MDV proteins has hindered the progress in such studies. This prompted us to develop pipelines to tag MDV genes as an alternative method for this purpose. Here we describe the application of CRISPR/Cas9 gene-editing approaches to tag the phosphoprotein 38 (pp38) gene of the MDV vaccine strain CVI988 with both V5 and green fluorescent protein (GFP). This rapid and efficient viral-gene-tagging technique can overcome the shortage of specific antibodies and speed up the MDV gene function studies significantly, leading to a better understanding of the molecular mechanisms of MDV pathogenesis.

## 1. Introduction

Marek’s disease virus (MDV), the causative agent of MD, is an acutely transforming, cell-associated avian herpesvirus belonging to the genus *Mardivirus*. MDV is highly contagious, and chicks get infected via the respiratory route through inhalation of poultry dust from contaminated poultry houses. Once infected, MDV undergoes a cytolytic infection involving different cell populations, including B and T lymphocytes, when high levels of expression of most viral genes are observed [1,2,3]. The cytolytic infection cycle, which lasts for up to 7 days, switches to latent infection when most of the viral gene expressions are shut off. Some of the latently infected CD4+ T cells in susceptible unvaccinated birds subsequently get transformed into neoplastic cells, resulting in the development of lymphomas, leading to high levels of mortality [4,5]. Extensive studies in recent years have identified some of the major viral proteins that contribute directly to the neoplastic transformation and development of tumors. These included the major oncoprotein Meq [6,7,8,9], MDV-encoded microRNAs [10], the virus-encoded telomerase RNA (vTR) [11], and viral telomeric repeats (TMRs) [2,12]. While these studies have no doubt provided insights into the direct determinants of neoplastic transformation, the roles of the majority of other viral proteins, which may also be critical to the understanding of viral pathogenicity, need to be explored.

Previous studies demonstrated the important role of MDV genes involved in MD pathogenesis, including the genes located in the long repeat regions of the genome, such as *Meq* [9,13,14], pp38 [15,16], viral telomerase RNA [11,17], viral IL-8 [18,19,20,21], and RLORF4 [22], as well as the genes in the unique long (UL) region, such as the large subunit of the ribonucleotide reductase (RR) enzyme [23] and VP22 protein encoded by UL49 [24]. All of these were achieved via mutational analysis using either cosmid DNA or bacterial artificial chromosome (BAC) technologies. Although the application of BAC technology has opened new avenues in basic herpesvirus research, cloning of viral genomes as a BAC plasmid is time consuming and has not been possible with many virus strains. In contrast, the latest CRISPR/Cas9 editing system allows for much simplified and efficient gene editing in many settings, including genome manipulation of several large DNA viruses, such as herpes simplex virus type I [25], pseudorabies virus [26,27,28,29], vaccinia virus [30], Kaposi’s sarcoma-associated herpesvirus [31], human cytomegalovirus [32,33], Epstein–Barr virus [34], guinea pig cytomegalovirus [35], duck enteritis virus [36], herpesvirus of turkeys [37,38], and Marek’s disease virus [39,40,41].

CRISPR/Cas9-based gene editing on the MDV genome has given opportunities to speed up the studies of gene function and identification of pathogenic determinants. The first study to demonstrate effective use of the CRISPR/Cas9 system on the MDV genome involved the generation of a Meq and pp38 deletion mutant of the MDV serotype 1 vaccine strain CVI988/Rispens in MDV-infected CEF using the double-gRNA transfection/virus infection strategy [41]. Using the same approach, deletion mutants of a single or a cluster of MDV-encoded miRNAs in MDV-1 strain RB-1B was successfully achieved [42]. Subsequently, editing of the MDV genome using the CRISPR/Cas9 system was extended to the integrated viral genomes of MDV-transformed lymphoblastoid cell lines. Deletion of the pp38 gene from the MDV genome of MDV transformed cell lines (MDCC-MSB-1 and MDCC-HP8 cells) showed an increase in the proliferation of the edited cells, indicating that pp38 is not necessary for the transformation of T lymphoma cell lines [39]. Similarly, successful deletion of miR-M4 demonstrated that the expression of the MDV-miR-M4 gene is not essential for the maintenance of the transformed state of the MDCC-HP8 tumor cell line, despite its known critical role in the induction of MD lymphomas [40]. More recently, the CRISPR/Cas9 approach was used for targeting essential MDV genes to block MDV replication in cultured cells [43]. Another study demonstrated significant in vivo inhibition of MDV by expressing Cas9 and gRNA against ICP4 in transgenic chickens [44].

Although CRISPR/Cas9 editing has been increasingly used in MDV gene function and host–virus interaction studies, thus far, only gene disruption/deletion has been applied in MDV gene editing using one or more gRNAs. The physical detection of viral gene expression and details of cellular localization of the viral proteins are valuable for host–virus interaction studies during virus replication and the progression of the disease development. However, the unavailability of antibodies against most of the viral genes has significantly hampered the progress of such studies. Targeted gene editing using CRISPR/Cas9 facilitates the introduction of donor DNA at specific loci in the genome, allowing for tagging proteins of interest with commonly used protein tags [45,46]. MDV-encoded phosphoprotein pp38 is essential for the lytic infection of B cells, while it was dispensable for lytic infection of the feather follicle epithelial cells or the induction of tumors [47]. pp38 is also widely considered as a biomarker for the lytic switch of infection in MDV transformed lymphoblastoid cell lines (LCLs) [48]. Our recent work showed that the activation of pp38 using a CRISPRa system induces lytic replication in LCLs [49]. The availability of the pp38-specific antibody BD1 allowed us to compare the detection result between BD1 and anti-V5 antibodies and hence assess the feasibility of viral gene tagging using the CRISPR/Cas9 system. In the present study, we developed a rapid and efficient method to tag the pp38 of the MDV vaccine strain CVI988 with V5 and GFP using the CRISPR/Cas9 editing system as an alternative to the detection using antibodies specific to viral proteins. The expression of the tagged pp38 was easily detected with either anti-V5 antibody for V5-tagged pp38, where the same result was obtained with pp38-specific antibody BD1, or monitored with IncuCyte S3 for GFP tagged pp38 during virus replication. These results showed that tagging viral genes in the MDV genome using CRISPR/Cas9 editing can be a valuable method for the detection of viral gene expression in infected cells, particularly when specific antibodies are not available.

## 2. Materials and Methods

### 2.1. Cell Culture and Viruses

Primary chick embryo fibroblasts (CEF) were prepared from 10-day-old specific-pathogen-free (SPF) embryos and maintained in M199 medium (Thermo Fisher Scientific, Daneshill, Basingstoke, UK) supplemented with 5% fetal bovine serum (FBS, Sigma, Gillingham, Dorset, UK), 100 units/mL of penicillin and streptomycin (Thermo Fisher Scientific), and 10% tryptose phosphate broth (Sigma). Commercial CVI988 vaccine stock Cryomarex Rispens was obtained from Boehringer Ingelheim (Boehringer Ingelheim, Ingelheim, Rhineland-Palatinate, Germany).

### 2.2. Construction of sgRNA and Donor Template

The gRNA-targeting C-terminal of pp38 of CVI988 genome was designed using CRISPR guide RNA-designing software (http://crispor.tefor.net/, accessed on 24 October 2019) and cloned into the CRISPR/Cas9 vector pX330A-1×2 by introducing the synthesized oligo-DNA primers pp38-gRNA-F and pp38-gRNA-R corresponding to the target sequence into *Bbs*I restriction sites. A single-stranded donor DNA of TTGTCGTTGGGGGAGTGGATTCTGGGGAGGTGGAATCTGGAGAAACAAAATCTGAATCAAATGGTAAGCCTATCCCTAACCCTCTCCTCGGTCTCGATTCTACGTAAATTTAATACAGTGTAGCCGTACCCGACGTTGAAGGCGGAGATTAAGCGAATTCTCACCTTTACGAATATTGGTGCAGACAAAGACCAAAAAAT harboring Protospacer adjacent motif (PAM)-blocking mutations and V5 tag coding sequence flanked by a 62 bp left homology arm (LHA) and 96 bp right homology arm (RHA) for V5 tagging was synthesized using integrated DNA technologies (IDT, Sheffield, South Yorkshire, UK). Double-stranded donor DNA harboring PAM-blocking mutations and a GFP coding sequence (amplified with primers pp38-GFP-B-F and pp38-GFP-B-R), flanked with 517 bp LHA (amplified with primers pp38-GFP-A-F and pp38-GFP-A-R) and 515 bp RHA (amplified with primers pp38-GFP-C-F and pp38-GFP-C-R) for GFP tagging, was generated by overlapping PCR with primers pp38-GFP-A-F and pp38-GFP-C-R for joining the LHA, GFP, and RHA sequences together. The oligonucleotides used are listed in Table 1.

### 2.3. Generation and Characterization of V5- and GFP-Tagged CVI988 Viruses 

A NEPA21 Electroporator was used for the transfection of CVI988 genomic DNA, gRNA plasmid, and donor DNA into CEF cells. Briefly, CEF cells were resuspended in 100 μL Opti-MEM medium (Thermo Fisher Scientific) and mixed with 2.5 μg of gRNA plasmid DNA, 2.5 μg of donor DNA, and 5 μg of CVI988 genomic DNA, and then electroporated with an optimized condition at voltage 250 V and a pulse width 1.5 ms of poring pulse. At 96 h post electroporation, single plaques from the V5-tagging were moved to a 24-well plate pre-seeded with CEFs and analyzed after 4 days of incubation. For GFP tagging, single GFP plaques were moved at 96 h post electroporation to a 24-well plate pre-seeded with CEFs followed by single live GFP cell sorting and analysis using PCR of the formed plaques following incubation. The harvested cells for PCR analysis were lysed in 1× Proteinase-K-based DNA extraction buffer (10 mM Tris-HCl, pH 8, 1 mM EDTA, 25 mM NaCl, and 200 µg/mL Proteinase K) at 65 °C for 30 min. A total of 2 µL of the extracted DNA was used as a template for the PCR with primers pp38-tag-F and pp38-tag-R (Table 1) outside the targeting site to identify the insertion of V5 or GFP. Subsequently, the PCR products were purified and sequenced.

### 2.4. Immunofluorescence Assay (IFA)

The expression of pp38 tagged with V5 in the mutant-virus-infected cell was evaluated using IFA. The CEF cells grown in 24-well plates were infected with the mutant and parental CVI988 viruses for 48–72 h. After fixing with 4% paraformaldehyde and permeabilizing with 0.1% Triton X-100, the cells were stained with primary mAb BD1 and secondary Alexa Fluor^TM^ 568 goat anti-mouse IgG_2a_ antibody for pp38 expression and primary mouse anti-V5 antibody (BIO-RAD) and secondary Alexa Fluor^TM^ 488 goat anti-mouse IgG antibody for V5/pp38 expression. Images were taken using IncuCyte S3 scanning microscope with 10× objective. For a stability check, images were taken from 36 separate regions per well covering approximately 70% of the well in 24-well plates. Only one out of 36 regions for each virus is shown in the results.

Infected CEF cells on the coverslips in 24 well plates were fixed with 4% chilled paraformaldehyde and permeabilized with 0.1% Triton X-100. The cells were then labeled with anti-heat shock protein 60 (HSP60) polyclonal antibody followed by goat anti-rabbit IgG labeled with Alexa Fluor 568 for HSP60 expression, anti-pp38 mAb BD1 followed by goat anti-mouse IgG labeled with Alexa Fluor 633 for pp38 expression, and anti-V5 antibody followed by rabbit anti-mouse IgG labeled with Alexa Fluor 488 for V5 expression. Coverslips were mounted in Vectashield onto microscope slides after cell nuclei staining with 4′,6-diamidino-2-phenylindole (DAPI). Images were taken using a Leica Stellaris 5 confocal laser scanning microscope (Leica Microsystems, Milton Keynes, Buckinghamshire, UK).

### 2.5. Single-Cell Sorting 

For single-cell cloning, the cells were washed twice with PBS containing 1% FBS and centrifuged at 450× *g* for 5 min at room temperature. The cell pellets were resuspended in cold PBS/5% FBS and single live GFP cells were sorted into a flat-bottom 96-well plate with growth medium by fluorescence-activated cell sorting (FACS) using FACSAria II (BD Biosciences, Wokingham, Berkshire, UK). 

### 2.6. Western Blotting

The expression of pp38 in CVI988-pp38-V5 virus was determined via Western blotting analysis using anti-pp38 mAb BD1 or anti-V5 antibody as the primary antibody. Briefly, 1 × 10^6^ infected cells were collected and boiled with TruPAGE^TM^ LDS sample buffer (Sigma) for 10 min. The samples were separated on a 4–12% TruPAGE^TM^ Precast Gel, and the resolved proteins were transferred onto PVDF membranes. Immunoblots were blocked with 5% skimmed milk and then incubated with anti-pp38 antibody or anti-V5 antibody. After probing with primary antibodies, the blots were incubated with secondary antibody IRDye^®^680RD goat anti-mouse IgG (LI-COR, Cambridge, Cambridgeshire, UK) and visualized using Odyssey Clx (LI-COR).

### 2.7. Live Cell Imaging of CVI988-pp38-GFP Growth

The growth of CVI988-pp38-GFP was monitored using IncuCyte S3 live-cell imaging (Sartorius, Royston, Hertfordshire, UK). Briefly, 1.6 × 10^5^ CEFs were seeded in a 24-well plate and infected with the CVI988-pp38-GFP virus (MOI 0.01). Images were captured every 4 h for 112 h from 36 separate regions per well using a 10× objective lens. Images of one region from all time points were exported to generate a movie.

### 2.8. Stability of the Inserted Tags in the Recombinant Viruses

The recombinant viruses CVI988-pp38-V5 and CVI988-pp38-GFP were passaged in CEF cells 15 times. The stability of each inserted tag was monitored using PCR and IFA. PCR was performed with primer pairs located at the flanking region of the insertion site (pp38-F and pp38-R) using DNA extracted from every 5th passage. IFA was performed to check the pp38 and V5 expressions every 5th passage following the method mentioned in Section 2.4.

## 3. Results

### 3.1. Generation of V5- and GFP-Tagged pp38 of CVI988 Virus Using CRISPR/Cas9 System

**Figure 1:**
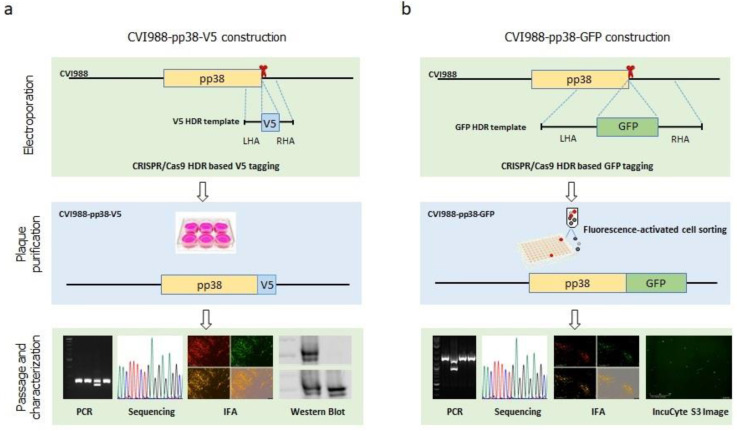
Strategy for the construction of pp38-tagged CVI988 viruses. (**a**) Generation of the CVI988-pp38-V5 virus. The V5 tag was inserted at the C-terminus of pp38 via HDR-CRISPR/Cas9. The synthesized donor template contained the V5 coding sequence and the homology arms at both sides. When the plaques were observed following the electroporation of the gRNA/Cas9 expression plasmid, synthesized V5 HDR template, and CVI988 genomic DNA into CEF, plaque purification was performed by moving the single plaque into fresh CEF. The purified plaques were then characterized using PCR, sequencing, IFA, and Western blotting, as described in the main text. (**b**) The generation of CVI988-pp38-GFP virus. The GFP sequence was inserted at the C-terminus of pp38 via HDR-CRISPR/Cas9. The donor template containing the GFP coding sequence with the homology arms at both sides was obtained using overlapping PCR. Plaque purification was performed using fluorescence-activated cell sorting. The purified plaques were characterized using PCR, sequencing, and IFA. The dynamics of the GFP-tagged virus growth were also monitored using IncuCyte imaging.

Previously, we successfully inserted the foreign sequences into both HVT and MDV genomes using the non-homologous end-joining (NHEJ) repair pathway. We now explored a strategy to tag the protein of interest with either the small peptide-coding sequence V5 or the fluoresent protein-coding sequence of GFP using a homology-directed repair (HDR) pathway with pp38 as an example for gene function studies. The gRNAs targeting the pp38 C-terminal were designed using the CRISPR guide RNA designing software. The gRNA closest to the stop codon of pp38 and with high scoring was chosen and cloned into the CRISPR/Cas9 vector pX330A. For V5 tagging, a synthesized single-stranded DNA donor harboring PAM-blocking mutations was used. For the GFP tagging, double-stranded DNA donor with a PAM-blocking mutation and GFP coding sequence flanked with 517 bp LHR and 515 bp RHA was generated using overlapping PCR. Previously, we developed the pipeline for efficient MDV/HVT genome editing, including deletion, insertion, and disruption with the NHEJ pathway. The pipeline involves transfection of the components required for editing of the target sequences, such as gRNA, Cas9, and the donor template, followed by infection of the viruses to be edited and subsequent plaque purification and characterization of the edited viruses. Considering the lower efficiency of the HDR pathway compared with the high efficiency of the NHEJ pathway, we explored the new strategy via the cotransfection of components required for editing and genomic DNA of the virus-infected cells to avoid the high background of the unedited viruses caused by virus infection. The detailed procedure for the generation and characterization of the pp38 fusion protein of the MDV vaccine strain CVI988 with either a V5 or GFP tag is described in Figure 1. Briefly, CEFs were electroporated with a mixture of gRNA expression plasmid, donor template, and CVI988 genomic DNA extracted from CVI988-infected CEFs. For V5 tagging, single plaques were moved to a 24-well plate seeded with CEFs after observing plaques. For GFP tagging, GFP plaques were moved to a 24-well plate, followed by single live GFP cell sorting into a 96-well plate for plaque purification. The isolated plaques were then subject to characterization using PCR, sequencing, IFA, and Western blotting or IncuCyte S3 imaging.

### 3.2. Characterization of CVI988-pp38-V5 and CVI988-pp38-GFP Viruses

**Figure 2:**
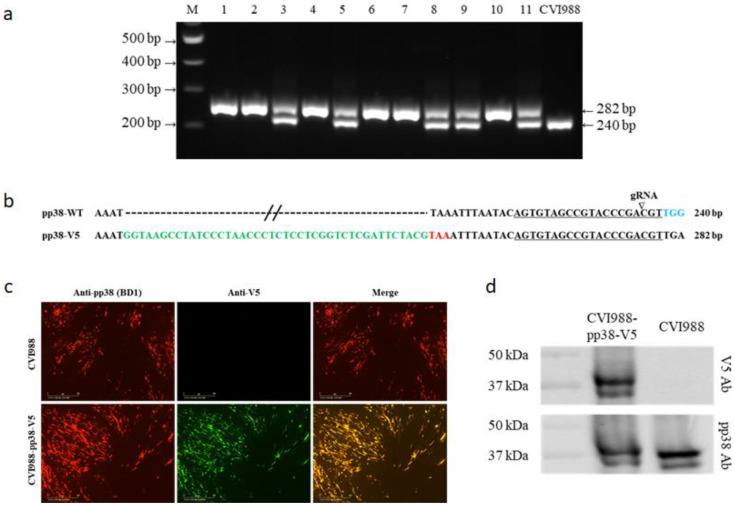
Generation and characterization of the CVI988-pp38-V5 virus. (**a**) PCR amplification of the edited region using primers pp38-tag-F and pp38-tag-R on the cell lysate of 11 isolated plaques and the parental CVI988 virus. The 282 bp top band represents the sequence containing V5. The 240 bp band represents the sequence without V5 insertion. (**b**) The sequences of the edited region of the 282 bp PCR product show the successful insertion of the V5 tag (green). The target sequence is underlined and the cleavage site is indicated by an arrow. The PAM sequence is in blue. The PAM-blocking mutation of TGG to TGA is shown using a color change. (**c**) Confirmation of successful V5 tagging of pp38 in infected CEF using IFA with anti-V5 monoclonal antibody (green) and anti-pp38 monoclonal antibody (red). Scale bar: 400 μm. (**d**) Detection of pp38-V5 expression in CVI988-pp38-V5 virus-infected CEF using Western blotting with the anti-V5 monoclonal antibody or anti-pp38 monoclonal antibody.

Eleven isolated plaques were analyzed for insertion of the V5 tag. To confirm the V5 sequence was inserted at the C-terminus of pp38 in frame with pp38, 5 × 10^4^ cells were lysed for DNA extraction and subjected to direct PCR using primers pp38-tag-F and pp38-tag-R located at the flanking region of the insertion site. As shown in Figure 2a, DNA from the parental virus-infected cells generated a 240 bp PCR product that corresponded to the unedited pp38. In contrast, six plaques contained a single 282 bp product that corresponded to the V5 insertion in pp38, representing the purified plaques with only the mutant viruses with the desired insertion. The remaining five plaques contained both 240 bp and 282 bp products, indicating the presence of a mixed population of both edited and unedited viruses. The sequence analysis of the edited band confirmed that the V5 tag (in green) was inserted in pp38 before the stop codon (in red) (Figure 2b). For further confirmation of proper expression of the V5 tagged pp38, virus-infected CEFs were examined using IFA with pp38-specific mAb BD1 and mouse anti-V5 mAb. As expected, pp38 in both CVI988 and CVI988-pp38-V5 was detected by the BD1 antibody, whereas it was only detected by the anti-V5 antibody in CVI988-pp38-V5-infected cells. More importantly, the confocal microscopic study revealed that anti-V5 staining and anti-pp38 staining overlapped, indicating both antibodies targeted the same protein (Figure 2c). We also examined the pp38 expression in CVI988-pp38-V5 virus-infected CEF via Western blotting analysis using both BD1 and anti-V5 antibodies. CEFs infected with parental CVI988 were used as the control. Similar to the IFA result, pp38 expression was detected with the BD1 antibody in both V5-tagged and untagged CVI988, whereas pp38 expression was only observed in CVI988-pp38-V5-infected CEFs but not in the parental CVI988-infected CEFs when the V5 antibody was used (Figure 2d).

**Figure 3:**
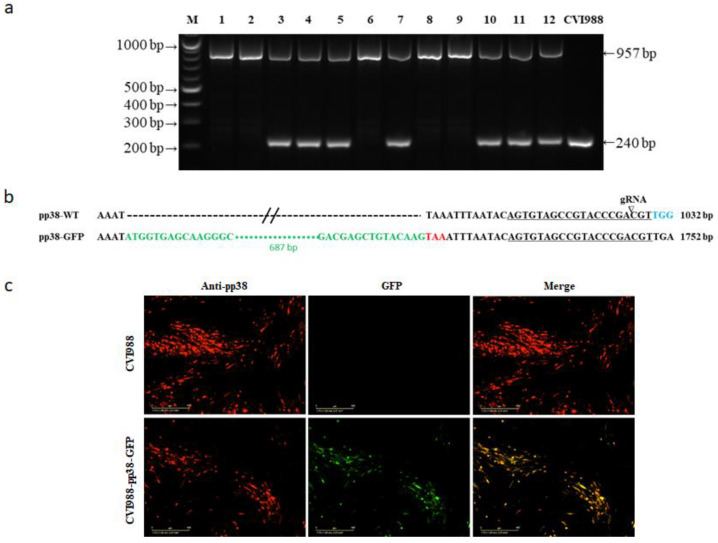
Generation and characterization of the CVI988-pp38-GFP virus. (**a**) PCR amplification of the edited region using primers pp38-tag-F and pp38-tag-R on the cell lysate of 12 single GFP cell-sorted plaques. The 957 bp top band represents the sequence containing GFP. The 240 bp band represents the sequence without the GFP insertion. (**b**) The sequences of the edited region of 957 bp PCR product show the successful insertion of GFP (green). The target sequence is underlined and the cleavage site is indicated by an arrow. The PAM sequence is in blue. The PAM-blocking mutation of TGG to TGA is shown using a color change. (**c**) Confirmation of successful GFP tagging of pp38 in infected CEF using IFA with anti-pp38 monoclonal antibody (red). Scale bar: 400 μm.

We analyzed 12 GFP-positive plaques obtained after sorting. To examine the purity of these plaques, DNA was extracted and PCR was performed using the primers pp38-tag-F and pp38-tag-R. Again, a 240 bp PCR product was generated from the parental CVI988 virus-infected cells, as expected (Figure 3a). Similar to the V5 tagging, two populations were present from the potential edited viruses. Five plaques with a single 957 bp product that corresponded to the GFP insertion in pp38 represented the purified plaques, with only the mutant viruses having the desired insertion. The other seven plaques had both bands, representing a mixed population of both GFP-inserted and unedited viruses. The insertion of the GFP sequence (in green) before the stop codon (in red) of pp38 was confirmed via sequencing of the 957 bp product of the purified virus (Figure 3b). After the infected cells were examined using IFA with pp38-specific mAb BD1, the expressed pp38 (red) overlapped with GFP, further confirming the precise tagging of pp38 with GFP (Figure 3c).

In addition to imaging the GFP-tagged infected cells directly using the fluorescence microscope, the introduction of the GFP tag allowed for imaging of the virus replication dynamics in living cells. To demonstrate this directly, we performed live-cell imaging of GFP-tagged virus infection in CEF using the IncuCyte S3 Live-Cell Imaging system (Supplementary Video S1). The video captured during the virus replication in real time showed the dynamics of the virus growth and plaque formation. This experiment showed that pp38-GFP-tagging of the MDV gene was compatible with live-cell imaging of viral protein dynamics during the course of virus replication.

### 3.3. Stability of CVI988-pp38-V5 and CVI988-pp38-GFP

**Figure 4:**
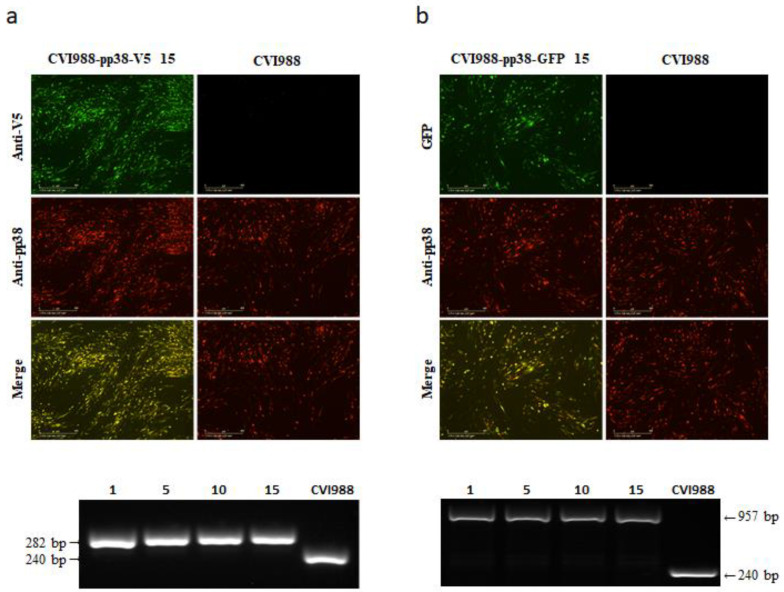
Stability of CVI988-pp38-V5 and CVI988-pp38-GFP viruses. (**a**) Top panel: CVI988-pp38-V5 virus-infected CEF at passage 15 was stained with anti-V5 monoclonal antibody (green) and anti-pp38 monoclonal antibody (red). Bottom panel: PCR to confirm the presence/absence of the V5 tag at the edited region using primers pp38-tag-F and pp38-tag-R on the cell lysate from CVI988-pp38-V5 virus infection at passages 1, 5, 10, and 15. The absence of a lower band in the CVI988-pp38-V5 virus during a passage indicates that the CVI988-pp38-V5 virus was stable. The sizes of the fragments are indicated. (**b**) Top panel: CVI988-pp38-GFP virus-infected CEF at passage 15 was stained with anti-pp38 monoclonal antibody (red). Bottom panel: PCR to confirm the presence/absence of the GFP at the edited region using primers pp38-tag-F and pp38-tag-R on the cell lysate from CVI988-pp38-GFP virus infection at passages 1, 5, 10, and 15. The absence of a lower band in the CVI988-pp38-GFP virus during a passage indicates that the CVI988-pp38-GFP virus was stable. The sizes of the fragments are indicated. Scale bar: 400 μm.

Previously, we showed that three foreign gene expression cassettes can be stably integrated into the HVT genome during repeated passages [38]. To determine the stability of V5/GFP-fused pp38 in the mutant CVI988 viruses during continuous passages, CVI988-pp38-V5 and CVI988-pp38-GFP were sequentially passaged in primary CEFs for 15 passages. The infected cells were examined using IFA and PCR every fifth passage. For IFA, the expression of V5-tagged pp38 was examined with anti-V5 antibody, together with anti-pp38 mAb BD1, to identify the double-stained cells demonstrating the stable integration of V5. For the CVI988-pp38-GFP virus, only pp38 staining with BD1 was needed, as GFP can be visualized directly under a fluorescence microscope. If the infected cells only show positive labeling with BD1 antibody, it indicates the loss of an inserted tag from the recombinant viruses during the passage. Figure 4 shows the staining results of one out of 36 regions in one well of the 24-well plate at the 15th passage. The presence of dual labeling in all of the infected cells demonstrated that the V5 tag (Figure 4a) and GFP tag (Figure 4b) were stably integrated into the CVI988 genome. To further confirm this, viral DNA was extracted and analyzed via PCR using primers located at the flanking region of the insertion site. As shown in the lower panels of Figure 4, the small bands amplified from CVI988 infection represent the sequence spanning the gRNA target site before the tag insertion, where the large bands in the tagged virus-infected cells were the expected size for the sequence containing the inserted V5 (Figure 4a)/GFP (Figure 4b) tag. The absence of the smaller bands in the recombinant virus-infected cells after repeated passages indicated that both the V5 and GFP tags were stably integrated into the CVI988 pp38 locus.

### 3.4. Possible Interaction of pp38 with Mitochondria Structure

**Figure 5:**
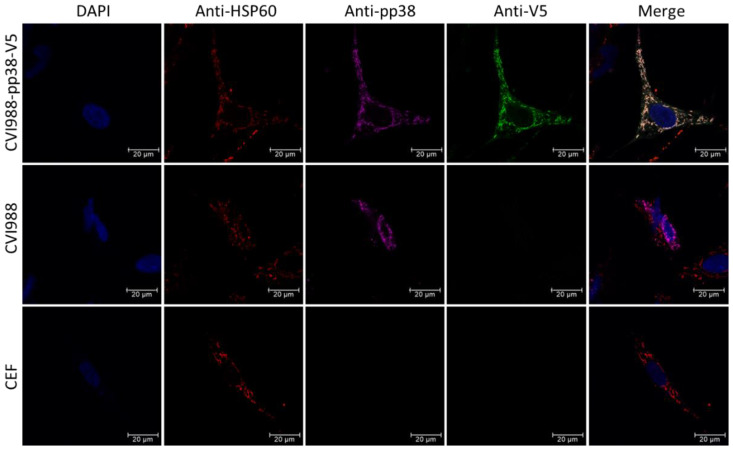
pp38 and 60 kDa heat shock protein (HSP60) were co-localized in the mitochondria. The expression of HSP60 and pp38 in CVI988-pp38-V5 virus-infected CEFs was detected with IFA. pp38 expression was detected with mAb BD1 followed by goat anti-mouse IgG labeled with Alexa Fluor 633 (far red). HSP60 expression was detected with anti-HSP60 polyclonal antibody followed by goat anti-rabbit IgG labeled with Alexa Fluor 568 (red). V5 expression was detected with anti-V5 mAb followed by rabbit anti-mouse IgG labeled with Alexa Fluor 488 (green). The nuclei were stained with DAPI (blue). Scale bar: 20 μm.

Previously, we observed the cytoplasmic distribution of the pp38-mCherry fusion protein in CVI988-infected CEF. Further studies using confocal microscopy suggested pp38 expression close to the mitochondria. To obtain a detailed understanding of pp38 subcellular localization and the potential interaction with the mitochondrial structures, we stained the CVI988-infected CEF with the anti-HSP60 antibody, an available chicken mitochondria marker antibody, pp38-specific mAb BD1 (purple), and V5 antibodies (green) (Figure 5). The parental CVI988-infected CEF and un-infected CEF were used as controls. The result showed that pp38 was colocalized with HSP60. The colocalization was observed as light orange. More importantly, the three colors were all overlapping, indicating the V5 antibody can be used as an alternative to pp38-specific mAb BD1.

## 4. Discussion

The MDV genome is approximately 180 kb long and encodes more than 100 genes that are involved in various processes of the viral lifecycle. The role of a majority of MDV-encoded proteins and host–virus interactions contributing to the MDV-induced oncogenesis are still to be explored. Following the successful application of the CRISPR/Cas9 system on HVT and MDV genome editing for gene function and virus–host interaction studies [39,40,41,42,50,51] and recombinant vaccine development [37,38,52] with a highly efficient NHEJ repair pathway, we report here the first use of the HDR repair pathway to tag the MDV viral gene pp38 of the vaccine strain CVI988 with V5 and GFP as an alternative of the antibody generation approach. The rapid and efficient tagging of the MDV viral gene provides huge opportunities for gene function studies without the need for generating specific antibodies against the viral proteins. To our knowledge, this is the first study to demonstrate the effective use of the CRISPR/Cas9 system in viral gene tagging of the MDV-1 genome for studies of viral gene function.

Two general repair pathways, namely, NHEJ and HDR [53,54], are involved in the repair of the double-stranded breaks (DSBs) created by Cas9 cleavage. The NHEJ repair pathway, which directly connects the cut ends leading to insertion/deletion (indel) mutations, is more efficient than the HDR repair pathway, as NHEJ occurs throughout the cell cycle [55], whereas HDR only occurs during the S and G2 phases [56,57,58]. So far, NHEJ is the only repair pathway utilized for gene deletion/insertion in MDV genome editing research [39,40,41,42,43,44]. While it is possible to knock-in DNA sequences at specific loci through CRISPR/Cas9-mediated NHEJ in the MDV genome [39], NHEJ-based editing is not precise and the junctions between donors and breakpoints are unpredictable. In contrast, HDR-mediated precise knock-in genome modifications are a more valuable approach for functional analysis, such as single nucleotide polymorphism (SNP) exchange and the insertion of small affinity tags or sequences, such as loxP elements [59]. Single-stranded DNA oligonucleotides (ssODN) or long double-stranded DNA (dsDNA) donors are commonly used as homology repair templates to achieve precise integration using HDR [60,61]. Considering the size of V5 (42 bp) and GFP (717 bp), the ssODN donor template for V5 tagging and dsDNA donor template for GFP tagging were used to tag pp38 as a proof of principle for MDV gene function study.

Previously, a transfection/infection methodology that involves transfection of Cas9/gRNA expression plasmids into CEF followed by infection of the parental virus, plaque purification, and characterization of the mutant viruses was used for gene deletion of replicating MDV/HVT in CEF cells with NHEJ repair [41,42,51]. Considering the likely high background of the wild-type virus with low efficient HDR-based editing of this approach, we developed a new pipeline for generating mutant MDV with precise mutation using co-electroporation of genomic DNA extracted from MDV-infected CEF, together with a Cas9/gRNA expression plasmid and the donor DNA, followed by an analysis of the formed plaques. Indeed, the approach was highly efficient, with a positive PCR band present in all analyzed plaques for both V5 (Figure 2a) and GFP tagging (Figure 3a). Moreover, 6 out of 11 (54.5%) for V5 tagging and 5 out of 12 (41.7%) for GFP tagging of purified MDV plaques were obtained from the first round of plaque screening, avoiding the need for further rounds of plaque purification. The mutant viruses with the GFP tag were relatively easier to obtain, as the fluorescent plaques could be visualized and identified under a fluorescence microscope, although the actual incorporating rate might be lower compared with V5. For non-fluorescent epitope tagging, such as with V5, this extremely efficient tagging system of the MDV gene could be a very useful tool for studies in MD pathogenesis and host–virus interactions. The factors contributing to the highly efficient V5 tagging could have been due to the position and high efficiency of chosen gRNA, small-sized insert, and high efficiency of asymmetrical ssDNA donor, as reported previously [62].

To test whether the HDR sequence modification was stably maintained in the MDV genome, both tagged viruses were passaged 15 times in CEF and tested using both IFA and PCR to observe the appearance of the wild-type viruses. The results showed that both V5 and GFP were stably integrated into the CVI988 genome, as there was no untagged virus after 15 passages. Although this is not surprising considering the relatively small size of the insert, as we showed previously that the HVT recombinant can accommodate three foreign gene expression cassettes [38], this is the first example to show the stable expression of a fusion protein in the MDV genome generated using the CRISPR/Cas9 system rather than a foreign gene being inserted in the intergenic region of the genome [37,38,52] or within the gene sequence where the gene expression is disrupted [39], providing the assurance of feasibility to tag the MDV genes using this approach.

The QT35 is a quail fibroblast cell line latently infected with MDV [63]. The potential role for pp38 in the mitochondrial dynamics and OxPhos pathway was suggested following the finding that the induction of pp38 from the QT35-derived cell line QTP32 expressing pp38 under the control of an inducible promoter resulted in a significant increase in mitochondrial succinate dehydrogenase activity and a decreased level of mitochondrial transcripts [64,65]. The availability of the mitochondria marker antibodies provided the opportunity to look at the possible interaction of the pp38 with the mitochondrial structures. Indeed, we observed the colocalization of pp38 with a mitochondria marker protein HSP60 using IFA. Although the detailed understanding of the interaction can be investigated further via high-resolution imaging using stimulated emission depletion (STED) microscopy and correlative light and electron microscopy (CLEM) to explore pp38′s potential roles in regulating metabolism and the OxPhos pathway, the colocalization result using different antibodies demonstrated that the anti-V5 antibody can be used for the functional analysis of pp38.

Overall, we present an efficient, precise, and broadly applicable strategy for MDV gene tagging for gene function studies using the CRISPR/Cas9-based homology-directed repair system. By knocking in an affinity/fluorescence tag at the gene of interest in the viral genome, the tagged gene can be studied by immunostaining (affinity tag) and live-cell imaging (fluorescence tag) to investigate the expression, subcellular distribution, host–virus interactions, and dynamic distribution of the tagged protein during virus replication. The current approach can be rapidly developed and expanded to any protein of interest in the MDV genome of both a lytic replicating virus and in MDV-1-transformed cell lines to study MDV gene function, pathogenesis, host–virus interaction, latency, reactivation, and molecular determinants of MDV oncogenicity.

## Figures and Tables

**Table 1:** Primers used for gRNA cloning, donor generation, and identification of the tagged pp38.

Primer	Sequence (5′–3′)
**pp38-gRNA-F**	CACCGGTGTAGCCGTACCCGACGT
**pp38-gRNA-R**	AAACACGTCGGGTACGGCTACACC
**pp38-tag-F**	GTTGGGGGAGTGGATTCTGG
**pp38-tag-R**	TCCTCGATTGGTGGGGAGAT
**pp38-GFP-A-F**	CACCGCGACCCGAGAGAAAGATCG
**pp38-GFP-A-R**	TCCTCGCCCTTGCTCACCATATTTGATTCAGATTTTGTTT
**pp38-GFP-B-F**	AAACAAAATCTGAATCAAATATGGTGAGCAAGGGCGAGGA
**pp38-GFP-B-R**	CTCCGCCTTCAACGTCGGGTACGGCTACACTGTATTAAATTTACTTGTACAGCTCGTCCA
**pp38-GFP-C-F**	TGGACGAGCTGTACAAGTAAATTTAATACAGTGTAGCCGTACCCGACGTTGAAGGCGGAG
**pp38-GFP-C-R**	CATCCGAGTGGTACGTGCAT

## Data Availability

Not applicable.

## Supplementary Materials

The following are available online at https://www.mdpi.com/article/10.3390/v14020436/s1, Video S1: Growth of CVI988-pp38-GFP virus monitored in real time using IncuCyte S3 live imaging system.
