# Supplementary material for: V5 and GFP Tagging of Viral Gene pp38 of Marek’s Disease Vaccine Strain CVI988 Using CRISPR/Cas9 Editing
Source: Viruses. 2022 Feb 21;14(2):436. doi: 10.3390/v14020436 (PMC8879161; doi:10.3390/v14020436)
Supplement: Supplementary file 1 [file viruses-14-00436-s001.zip › Supplementary video S1 legend.pdf]

Supplementary Video S1. Growth of CVI988-pp38-GFP virus monitored in real time using In-cuCyte S3 live imaging system. GFP cell phase object of CVI988-pp38-GFP virus was captured every 4h for 112 h from 36 separate regions per well by IncuCyte S3. Images of one region from all time points was exported to generate movie. The scale bar, 400 μm.
